# Quantitative susceptibility mapping is more sensitive and specific than phase imaging in detecting chronic active multiple sclerosis lesion rims: pathological validation

**DOI:** 10.1093/braincomms/fcaf011

**Published:** 2025-01-11

**Authors:** Kelly M Gillen, Thanh D Nguyen, Alexey Dimov, Ilhami Kovanlikaya, Ha Manh Luu, Emily Demmon, Daniel M Markowitz, Francesca Bagnato, David Pitt, Susan A Gauthier, Yi Wang

**Affiliations:** Department of Radiology, Weill Cornell Medicine, New York, NY 10065, USA; Department of Radiology, Weill Cornell Medicine, New York, NY 10065, USA; Department of Radiology, Weill Cornell Medicine, New York, NY 10065, USA; Department of Radiology, Weill Cornell Medicine, New York, NY 10065, USA; Department of Radiology, Weill Cornell Medicine, New York, NY 10065, USA; Department of Neurology, Weill Cornell Medicine, New York, NY 10065, USA; Department of Radiology, Weill Cornell Medicine, New York, NY 10065, USA; Department of Neurology, Nashville VA Medical Center, Tennessee Valley Healthcare System, Nashville, TN 37212, USA; Department of Neurology, Yale School of Medicine, New Haven, CT 06511, USA; Department of Neurology, Weill Cornell Medicine, New York, NY 10065, USA; Department of Radiology, Weill Cornell Medicine, New York, NY 10065, USA

**Keywords:** quantitative susceptibility mapping (QSM), phase, iron, inflammation, multiple sclerosis

## Abstract

Quantitative susceptibility mapping and phase imaging are used to identify multiple sclerosis lesions with paramagnetic rims that slowly expand over time and are associated with earlier progression to disability, decreased brain volume and increased frequency of clinical relapse. However, the presence of iron-laden microglia/macrophages at the lesion rim and demyelination within the lesion both contribute to phase and quantitative susceptibility mapping images. Therefore, simultaneous pathological validation is needed to assess accuracies in identifying iron-positive lesions. MRI was performed on 15 multiple sclerosis brain slabs; 32 lesions of interest were processed for myelin, iron and microglial markers. Three experienced readers classified lesions as rim positive or negative on quantitative susceptibility mapping and phase; these classifications were compared with Perls’ stain as the gold standard. All 10 of the quantitative susceptibility mapping-positive lesions had iron-positive rims on histology. Of the 16 phase-positive lesions, only 10 had iron-positive rims on histology. Using Perls’ stain as the ground truth, the positive predictive value was 100% for quantitative susceptibility mapping and 63% for phase; the negative predictive value was 95% for quantitative susceptibility mapping and 94% for phase. Post-mortem imaging results demonstrate that quantitative susceptibility mapping is a more reliable indicator of an iron-positive rim compared with phase imaging.

## Teaser

Gillen *et al*. compared the accuracy of quantitative susceptibility mapping (QSM) and phase imaging for detecting iron+ lesions in post-mortem multiple sclerosis brains using histopathology as the reference standard and demonstrated that QSM is a more reliable indicator of an iron+ rim compared with phase imaging.

## Graphical Abstract

**Graphical Abstract:**
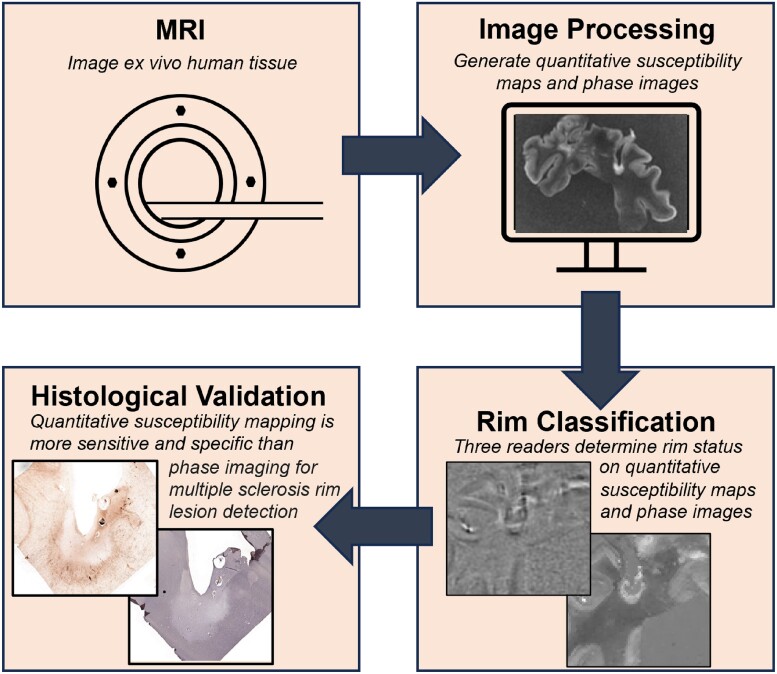

See Rovira and Pareto (https://doi.org/10.1093/braincomms/fcaf037) for a scientific commentary on this article.

## Introduction

Multiple sclerosis is a chronic autoimmune disease of the central nervous system characterized by inflammatory-demyelinating lesions and irreversible axonal and neuronal loss.^1^ Persistent smoldering inflammation and ongoing tissue damage in chronic active lesions have been implicated in progressive cognitive and ambulatory decline in multiple sclerosis,^2-4^ including more frequent clinical relapses and greater long-term relapse-independent disability progression.^5^ Paramagnetic rim lesions (PRLs) form a subset of chronic active lesions characterized by a dense rim of iron-laden pro-inflammatory microglia/macrophages on histopathology.^3,6-9^ PRL rim iron is an emerging diagnostic and treatment biomarker of the innate immune activity in multiple sclerosis,^10,11^ which can be identified *in vivo* by susceptibility-sensitive MRI methods including high-pass-filtered (HPF) phase imaging^8,12,13^ and quantitative susceptibility mapping (QSM).^14-17^

While both phase imaging and QSM can be used for PRL identification, prior studies have shown a higher prevalence of PRLs detected on the phase images compared with those on QSM.^16,18^ Physics theory^19^ and experimental phantom data^16,20^ have suggested that the rim appearance on HPF phase images can also arise from the field of magnetic sources inside the lesion core. The objective of this study was to validate the source of the PRL signal and establish PRL detection accuracy on phase and QSM images of *ex vivo* multiple sclerosis lesions using histology as the gold standard.

## Materials and methods

Fifteen formalin-fixed coronal brain slabs from 14 multiple sclerosis patients were obtained from the Rocky Mountain MS Center Tissue Bank (Table 1). Multiple sclerosis brain slabs were embedded in 1% agarose and scanned on 3T clinical MRI scanners (GE Healthcare, Milwaukee, WI, USA; SIEMENS, Erlangen) using the product head coil.^21^ The typical imaging protocol consisted of 2D T2-weighted fast spin echo sequence (voxel size = 0.5 × 0.5 × 1 mm^3^, TE = 64 ms, TR = 7.65 ms, readout bandwidth = 210 Hz/pixel, echo train length = 18, number of signal averages = 2, total scan time = 5 min 29 s) and 3D multi-echo gradient echo sequence (voxel size = 0.5 × 0.5 × 0.5 mm^3^, first TE = 3.4 ms, ΔTE = 5.6 ms, TR = 48 ms, number of echoes = 8, flip angle = 15°, readout bandwidth = 320 Hz/pixel, total scan time = 38 min 58 s) for QSM and phase maps. QSM images were reconstructed using a preconditioned total field inversion algorithm.^22^ Phase images were generated by applying a HPF to the phase of the complex data acquired at the TE closest to 20 ms to remove the large but smoothly varying background field from the phase image. Specifically, a 2D circularly symmetric low-pass Hanning kernel with a 128-voxel radius was multiplied with the k-space data and Fourier transformed to generate a low-pass-filtered complex image. The HPF phase image was obtained by taking the phase of the complex division between the original unfiltered image and the low-pass image.

**Table 1:** Patient demographics and disease course

Case #	Sex	Age	Disease duration (years)	Disease course
1	Male	46	9	RRMS
2	Male	80	Unknown	Stable, chronic
3	Female	74	31	SPMS
4	Female	60	Unknown	PPMS
5	Male	63	29	SPMS
6	Male	37	5	RRMS
7	Male	60	18	SPMS
8	Female	61	31	SPMS
9	Male	42	12	SPMS
10	Male	36	7	RRMS
11	Female	45	19	SPMS
12	Male	38	8	SPMS
13	Female	60	25	SPMS
14	Female	74	21	SPMS

PPMS, primary progressive multiple sclerosis; RRMS, relapsing-remitting multiple sclerosis; SPMS, secondary progressive multiple sclerosis.

White matter lesion hyperintense on T2FLAIR was classified as rim positive or negative on QSM (QSM+ or QSM−, respectively) and phase (Phase+ or Phase−, respectively) independently by three readers (I.K., F.B. and S.A.G.) blinded to lesion histology. There was a 2-week washout period between QSM and phase readings to avoid recall; reading sessions were then repeated to assess intra-rater agreement. The final rim status of each lesion was determined based on the initial classification for each reader and then the majority of the three individual readings.

Following MRI, lesions of interest were excised, embedded in paraffin and cut into 5-μm sections. Sections were further processed for histology and then incubated overnight with a primary antibody against myelin basic protein (MBP, Dako A0623, 1:500), CD68 (Cell Signaling #76437, 1:500) and inducible nitric oxide synthase (iNOS; Novus NB120-15203, 1:300) and a biotinylated secondary antibody and avidin/biotin staining kit with diaminobenzidine (DAB) as the chromogen (Vector Laboratories ABC Elite Kit and DAB Kit). To detect ferric iron, slides were immersed in 4% ferrocyanide/4% hydrochloric acid for 30 min in the dark. Staining was enhanced through incubation with DAB for 30 min at room temperature. After staining, all sections were rinsed, dehydrated, cover-slipped and digitized using a Mirax digital slide scanner (Molecular Cytology Core Facility, Memorial Sloan Kettering Cancer Center).

Independent of the readers for the MRI study, two additional readers (K.M.G. and D.P.) used MBP staining to confirm the presence of a multiple sclerosis lesion and then Perls’ stain to classify the lesion as either iron+ (iron present at the lesion periphery) or iron− (no iron present at the lesion periphery). There was complete agreement between both readers for iron rim classification.

### Statistical analysis

The Fleiss coefficient was calculated to measure inter-rater agreement in determining lesion rim status on QSM and phase images. Cohen’s *κ* was calculated for each of the three readers to measure the intra-rater agreement for determining lesion rim status on QSM and phase images for two reading sessions.

## Results

All 32 lesions had complete or near complete loss of myelin in the core, confirming their classification as multiple sclerosis white matter lesions. Of these 32 lesions, nine were classified as rim positive on both QSM and phase (QSM+/Phase+), seven were rim negative on QSM but rim positive on phase (QSM−/Phase+), one was rim positive on QSM but rim negative on phase (QSM+/Phase−), and 15 were rim negative on both QSM and phase (QSM−/Phase−) (Supplementary Table 1; Supplementary Fig. 1). The Fleiss coefficient for all three raters was moderate (0.57) for QSM rim status and slight (0.33) for phase rim status. Intra-rater agreement for QSM was 0.85 (almost perfect), 0.55 (substantial) and 0.79 (substantial) for each reader; for phase, intra-rater agreement was 0.44 (moderate), 0.63 (substantial) and 0.57 (substantial) for each reader, respectively.

**Figure 1:**
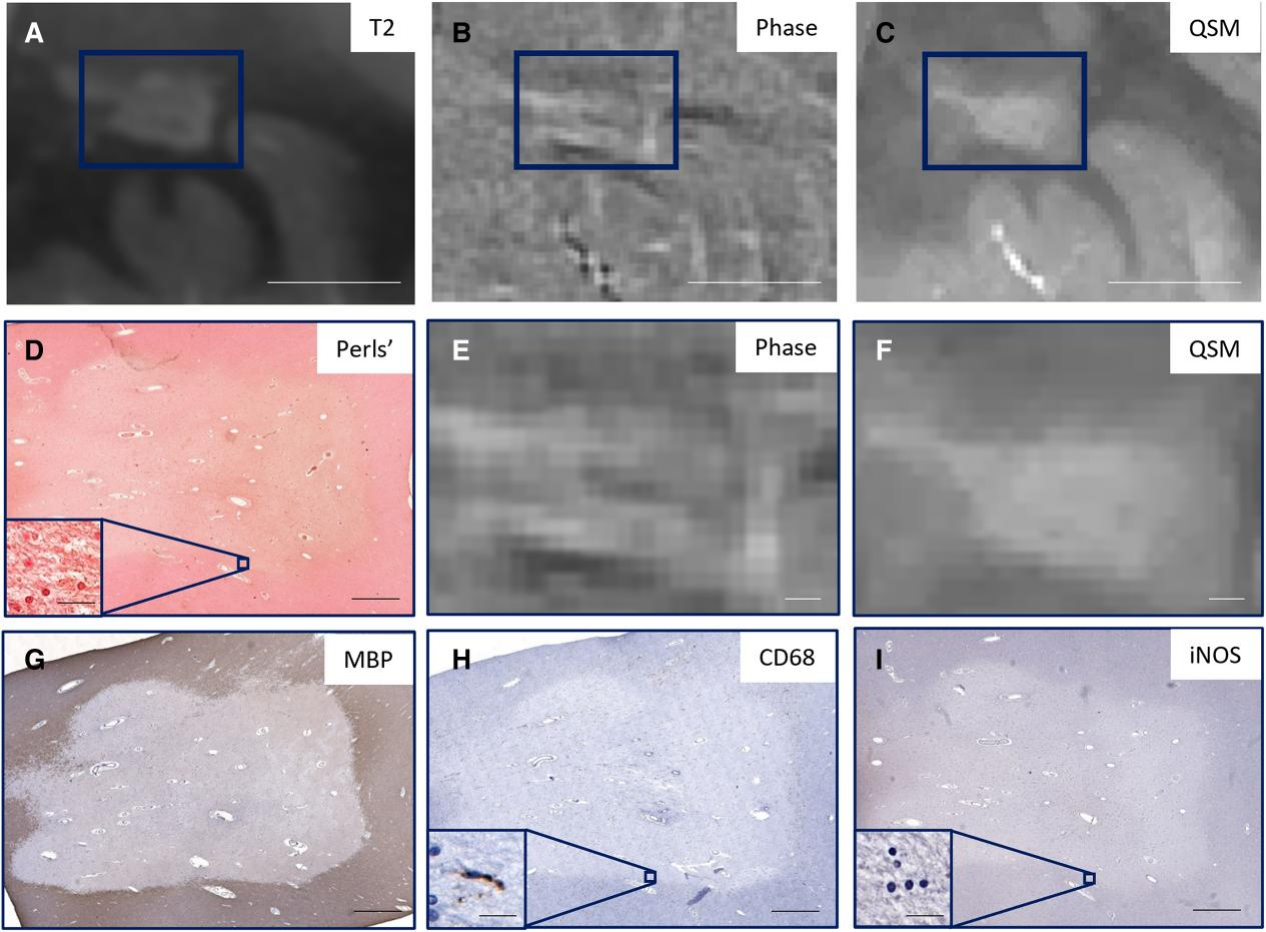
**Representative lesion that is iron− on Perls’ stain but classified as Phase+ and QSM−.** There are regions of hypointensity above and below the lesion on phase (**B** and **E**) but no corresponding hyperintensity on QSM (**C** and **F**), nor is there iron on Perls’ stain (**D**). (**A**) T2, (**B**) Phase and (**C**) QSM images of the lesion (rectangle) with higher magnification images of the lesion in (**E**) phase and (**F**) QSM with corresponding. (**D**) Perls’ to visualize iron, (**G**) MBP to visualize loss of myelin in the lesion centre, (**H**) CD68 to visualize microglia and macrophages and (**I**) iNOS to visualize activated microglia. (**A–C**) Scale bar = 1 cm; (**D–I**) scale bar = 1000 μm; (**D**, **H** and **I** insets) scale bar = 20 μm.

**Figure 2:**
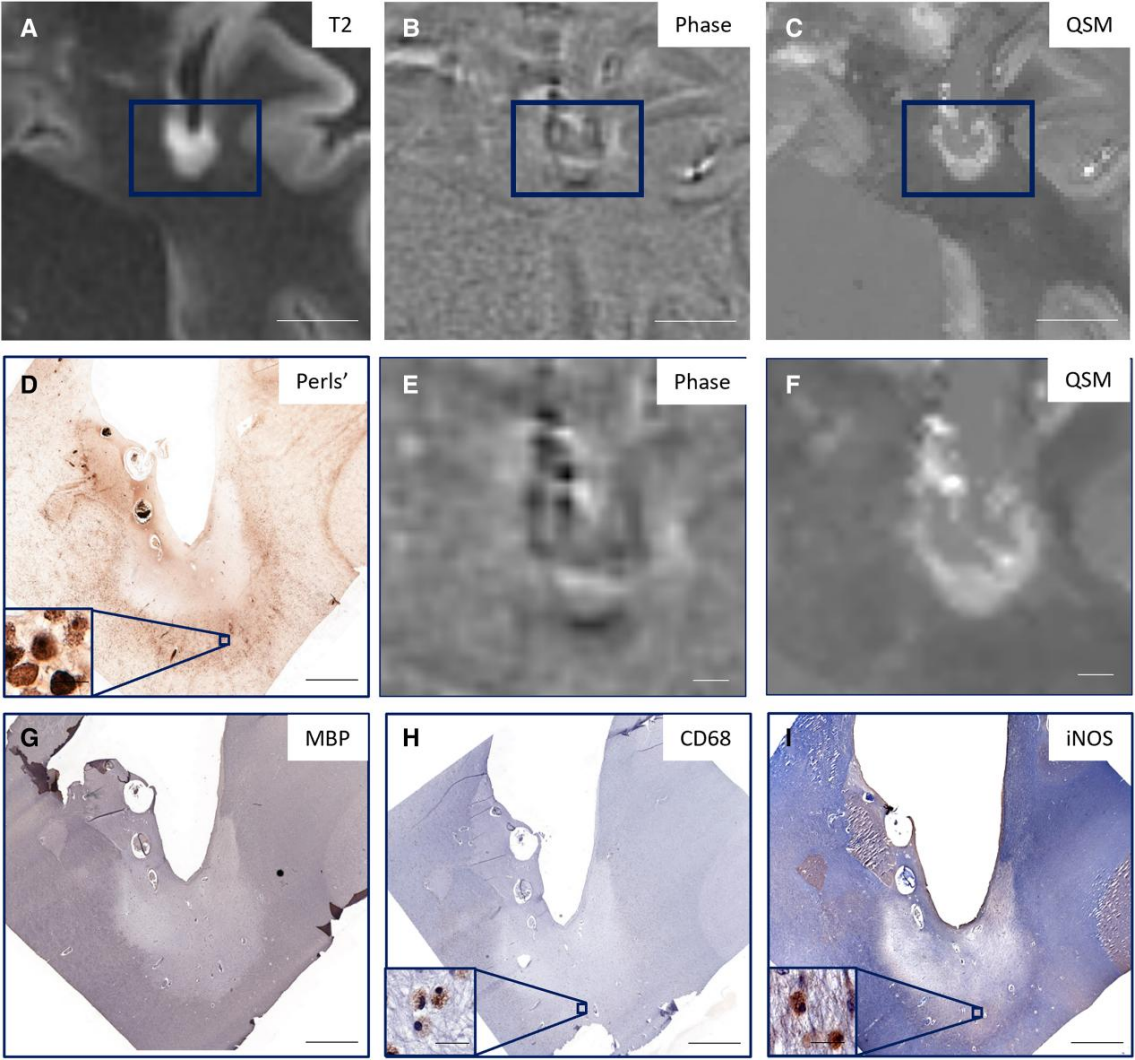
**Representative lesion that is iron+ on Perls’ stain and classified as Phase+ and QSM+.** There is a hypointense rim visible at the lesion periphery on phase (**B** and **E**) and a hyperintense rim visible on QSM (**C** and **F**), as well as *n* iron+ rim on Perls’ in **D**. (**A**) T2, (**B**) Phase and (**C**) QSM images of the lesion (rectangle); higher magnification of (**E**) phase and (**F**) QSM images, with corresponding (**D**) Perls’ to identify iron, (**G**) MBP to identify myelin, (**H**) CD68 to identify microglia and macrophages and (**I**) iNOS to visualize pro-inflammatory microglia. (**A–C**) Scale bar = 1 cm; (**D–I**) scale bar = 2000 μm; (**D**, **H** and **I** insets) scale bar = 20 μm.

**Figure 3:**
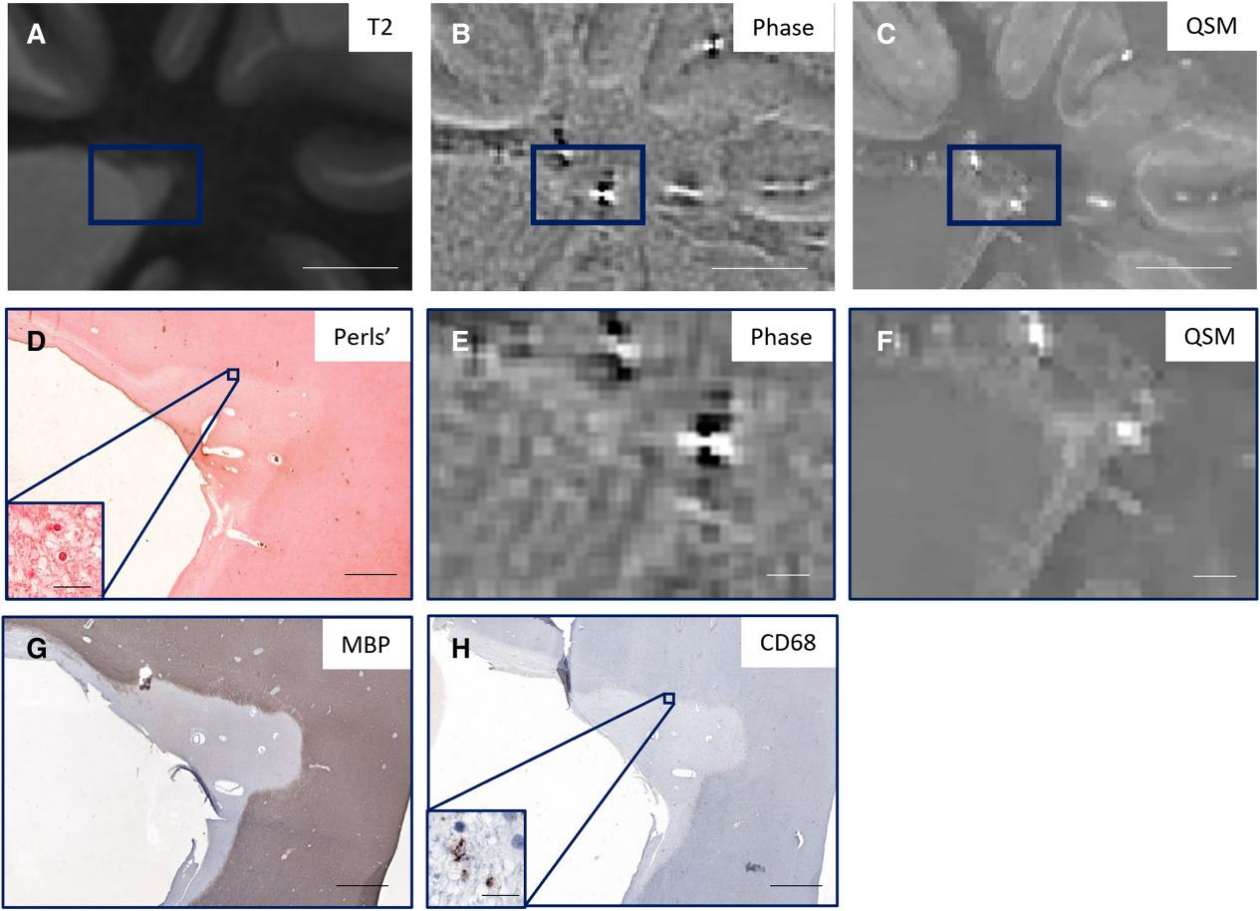
**Representative lesion that is iron− on Perls’ stain and classified as Phase− and QSM−.** There is no hyperintense rim visible on phase (**B** and **E**) and no hyperintense rim on QSM (**C** and **F**), nor is there iron on Perls’ (**D**). (**A**) T2, (**B**) Phase and (**C**) QSM images of the lesion (rectangle); higher magnification of (**E**) phase and (**F**) QSM images, with corresponding (**D**) Perls’ to visualize iron, (**G**) MBP to visualize myelin and (**H**) CD68 to visualize microglia and macrophages. (**A–C**) Scale bar = 1 cm; (**D–H**) scale bar = 2000 μm; (**D** and **H** insets) scale bar = 20 μm.

**Figure 4:**
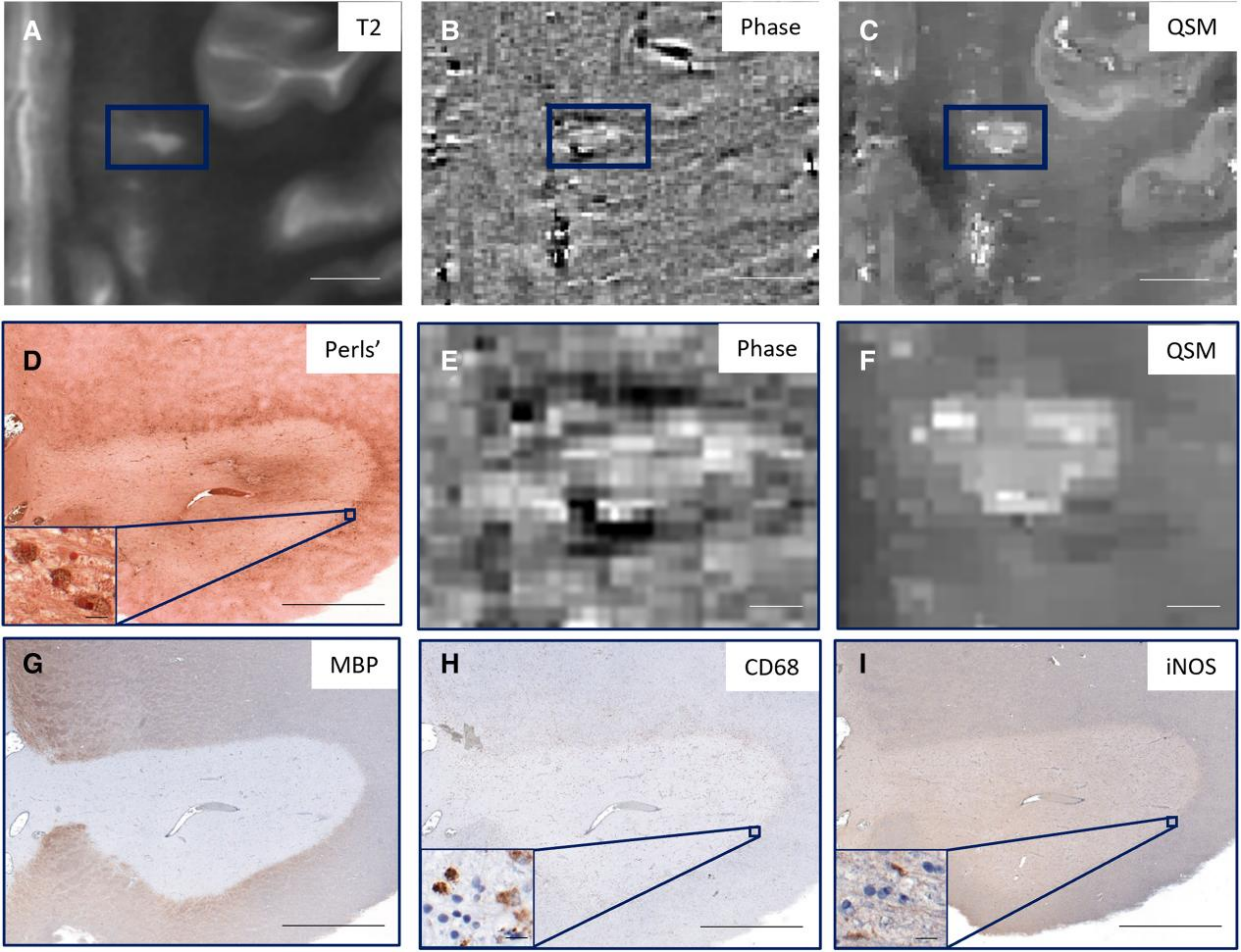
**Lesion that is iron+ on Perls’ stain but classified as Phase+ and QSM+.** The hypointense phase rim is visible above and below the lesion (**B** and **E**). Despite hyperintensity at the lesion periphery on QSM (**C** and **F**) and iron+ on Perls’ (**D**), this lesion was classified as Phase− and QSM−. (**A**) T2, (**B**) Phase and (**C**) QSM images of the lesion (rectangle); higher magnification of (**E**) phase and (**F**) QSM images, with corresponding (**D**) Perls’ to identify iron, (**G**) MBP to identify myelin and (**H**) CD68 for microglia/macrophages and (**I**) iNOS as an indicator of pro-inflammatory microglia. (**A–C**) Scale bar = 1 cm; (**D–I**) scale bar = 2000 μm; (**D**, **H** and **I** insets) scale bar = 20 μm.

Figure 1 shows an iron− lesion that was classified as Phase+ and QSM−. This lesion is uniformly hyperintense on QSM and contains few CD68 or iNOS-positive cells, indicating minimal microglia/macrophage activation. Figure 2 is representative of an iron+ lesion that was classified as Phase+ and QSM+ and that has activated microglia/macrophages (iNOS) at the lesion periphery. An iron− lesion classified as Phase− and QSM− is shown in Fig. 3; even though there is no iron present at the lesion periphery, there are CD68+ cells at the periphery. Figure 4 shows the only iron+ lesion that was classified as Phase+ but QSM−. This lesion has CD68+ and iNOS+ cells at the periphery, indicating the presence of activated microglia/macrophages. Although this lesion demonstrates hyperintensity at the rim, the readers may have conservatively labelled it as QSM− due to the prominent central vein.

There were a total of 11 iron+ lesions from four subjects. All 10 QSM+ lesions (100%) had iron rims on Perls’ (i.e. were iron+), while 10 out of 16 Phase+ lesions (62.5%) were iron+ on Perls’. Twenty-one QSM− lesions were iron− on Perls’, and one QSM− lesion was iron+ on Perls’. Fifteen Phase− lesions were iron− on Perls’, and one Phase− lesion was iron+ on Perls’ (Supplementary Table 1). Using the presence of iron on Perls’ as the ground truth, the positive predictive value was 100% for QSM and 63% for phase; the negative predictive value was 95% for QSM and 94% for phase. The sensitivity and specificity for QSM rim status were 91% and 100%, respectively; the sensitivity and specificity for phase rim status were 91% and 71%, respectively. The accuracy for QSM was 97%, while the accuracy for phase was 78%.

## Discussion

This was the first *ex vivo* validation study to directly compare QSM and HPF phase imaging for detecting iron+ multiple sclerosis lesions using histology as the gold standard. Our data show that QSM+ lesions with a hyperintense rim are more accurate than Phase+ lesions with a hypointense rim for indicating the presence of iron+ rims. In our study sample of 32 lesions, both methods provided high detection sensitivity (91%) for iron+ rims but QSM was found to be the superior technique with respect to specificity (100% versus 71%) and positive predictive value (100% versus 63%). As QSM can be readily obtained by applying dipole deconvolution to the phase data, our results suggest that QSM has the potential to become the standard MRI method for detecting multiple sclerosis lesions with iron+ rims in clinical practice.

Our findings are consistent with and provide pathological interpretation for prior reports indicating that some chronic multiple sclerosis lesions appear solid without a rim on QSM and show a rim on phase imaging.^4,16,20^ The discordance between QSM and phase imaging can be explained by the underlying physics whereby both solid and shell-shaped susceptibility distributions can produce a local field with a rim appearance on the HPF phase images,^16,19,20^ depending on lesion geometry, position relative to imaging plane^23^ and orientation with respect to the main field.^24^ For example, for a spherical lesion with a fully demyelinated core but without an iron+ rim, the unfiltered phase inside the sphere is uniform and outside the sphere is a dipole pattern, which is identical to a shell (rim).^19^ High-pass filtering required to remove the dominant background field in phase imaging nulls the uniform phase inside and generates a rim appearance, thus confounding rim interpretation.^16,20,25^ Furthermore, the solid susceptibility interior of a tissue often appears with confounding positive and negative lines on phase images.^25^ To avoid this misinterpretation of HPF phase, QSM utilizes dipole deconvolution^15^ and recovers local tissue susceptibility.^14,20^

It should be noted that imaging noise interferes with PRL interpretation. Phase imaging with necessary high-pass filtering tends to be noisier,^25^ which can make rim detection difficult. The inherent denoising in QSM improves conspicuity and consequent reader agreement: in this study, the Fleiss coefficient for all three raters was moderate (0.57) for QSM rim status and slight (0.33) for phase rim status. This moderate agreement may be because interpreting QSM images is unfamiliar to a radiologist or neurologist and therefore can be improved with reader training. For example, readers should be aware that the rim is not present around the entire lesion circumference, which may explain the misclassification as demonstrated in Fig. 4.

The presence of a paramagnetic rim has been proposed as a biomarker for the smoldering innate immune activity that occurs behind a sealed blood–brain barrier in chronic active multiple sclerosis lesions. *In vivo* positron emission tomography imaging using the 18-kDa translocator protein-binding radioligand ^11^C-PK11195, a marker of the activated microglia and macrophages, has shown that PRLs identified on QSM are associated with increased inflammation.^17^ While both phase imaging and QSM have proven useful for PRL identification, several important differences between the two approaches must be noted. First, a statistically significant association was found between clinical disability and a minimum lesion load of four PRLs by phase imaging.^5^ However, a recent study found a significant association with only one or more PRL on QSM.^26^ Second, in the same study, it was reported that lesions with a rim on QSM are associated with greater myelin damage compared with those without QSM rim (including those that have rim on the phase image). These findings in the *in vivo* multiple sclerosis brains are well supported by our results, which are histologically confirmed that phase imaging may overestimate the number of iron+ PRLs. Finally, unlike QSM, phase imaging is not quantitative and therefore cannot be used as a treatment biomarker for longitudinal changes in the innate immune activity in multiple sclerosis. QSM is a quantitative measure of susceptibility and can be used in longitudinal studies following subtle lesion changes over time to assess disease progression and/or response to treatment. In fact, a recent retrospective observational study evaluating the role of QSM for drug treatment has shown that multiple sclerosis patients treated with dimethyl fumarate, a drug that can penetrate into the brain, showed a greater reduction in susceptibility compared with those treated with glatiramer acetate.^10^ Additional studies on potential multiple sclerosis treatments targeting PRLs are ongoing,^27^ underscoring the potential use for QSM in providing a quantitative tool to assess treatment response.

There are several limitations to this study. Even though we sampled lesions from 15 multiple sclerosis patients, only a total of 32 lesions were analysed, with a variable number of lesions from each slab. Complete histology was not available for all lesions; some lesions lacked iNOS staining, but based on our previous work, we inferred that if a lesion was iron+, this iron was likely contained within pro-inflammatory microglia/macrophages.^9^ Furthermore, during reading sessions, the readers read all lesions on QSM and then all lesions on phase rather than randomly assessing both image types at baseline and then assessing the corresponding phase or QSM after the washout period. In this work, we did not perform a comparison of QSM and the echo-planar phase imaging sequence, which was used in several prior studies but is not available as a product on our scanners. Finally, iron rim depiction can be further improved using a combined R2* and QSM for separating iron from myelin.^28-30^

In conclusion, accurate determination of multiple sclerosis rim lesion status is important in assessing and quantifying disease treatment and progression. Our MR imaging with corresponding histology on post-mortem multiple sclerosis brain slabs indicates that QSM is a more reliable indicator of an iron+ rim than phase imaging.

## Supplementary Material

fcaf011_Supplementary_Data

## Data Availability

The data that support the fundings of this study are available from the corresponding author, upon reasonable request. Code is available for download at: https://pre.weill.cornell.edu/mri/pages/qsm.html.
