# Supplementary material for: Quantitative susceptibility mapping is more sensitive and specific than phase imaging in detecting chronic active multiple sclerosis lesion rims: pathological validation
Source: Brain Commun. 2025 Jan 11;7(1):fcaf011. doi: 10.1093/braincomms/fcaf011 (PMC11800486; doi:10.1093/braincomms/fcaf011)
Supplement: fcaf011_Supplementary_Data [file fcaf011_supplementary_data.docx]

**Supplementary Table 1.** Contingency tables for (A) QSM vs ground truth, (B) Phase vs ground truth, and (C) QSM vs Phase.

(A)

| **QSM** |  | **Ground truth (Perls’)** | |
| --- | --- | --- | --- |
|  |  | **Rim+** | **Rim-** |
| **Readers** | **QSM+** | 10 | 0 |
|  | **QSM-** | 1 | 21 |

(B)

| **Phase** |  | **Ground truth (Perls’)** | |
| --- | --- | --- | --- |
|  |  | **Rim+** | **Rim-** |
| **Readers** | **Phase+** | 10 | 6 |
|  | **Phase-** | 1 | 15 |

(C)

|  | **Phase+** | **Phase-** |
| --- | --- | --- |
| **QSM+** | **9** | **1** |
| **QSM-** | **7** | **15** |

**
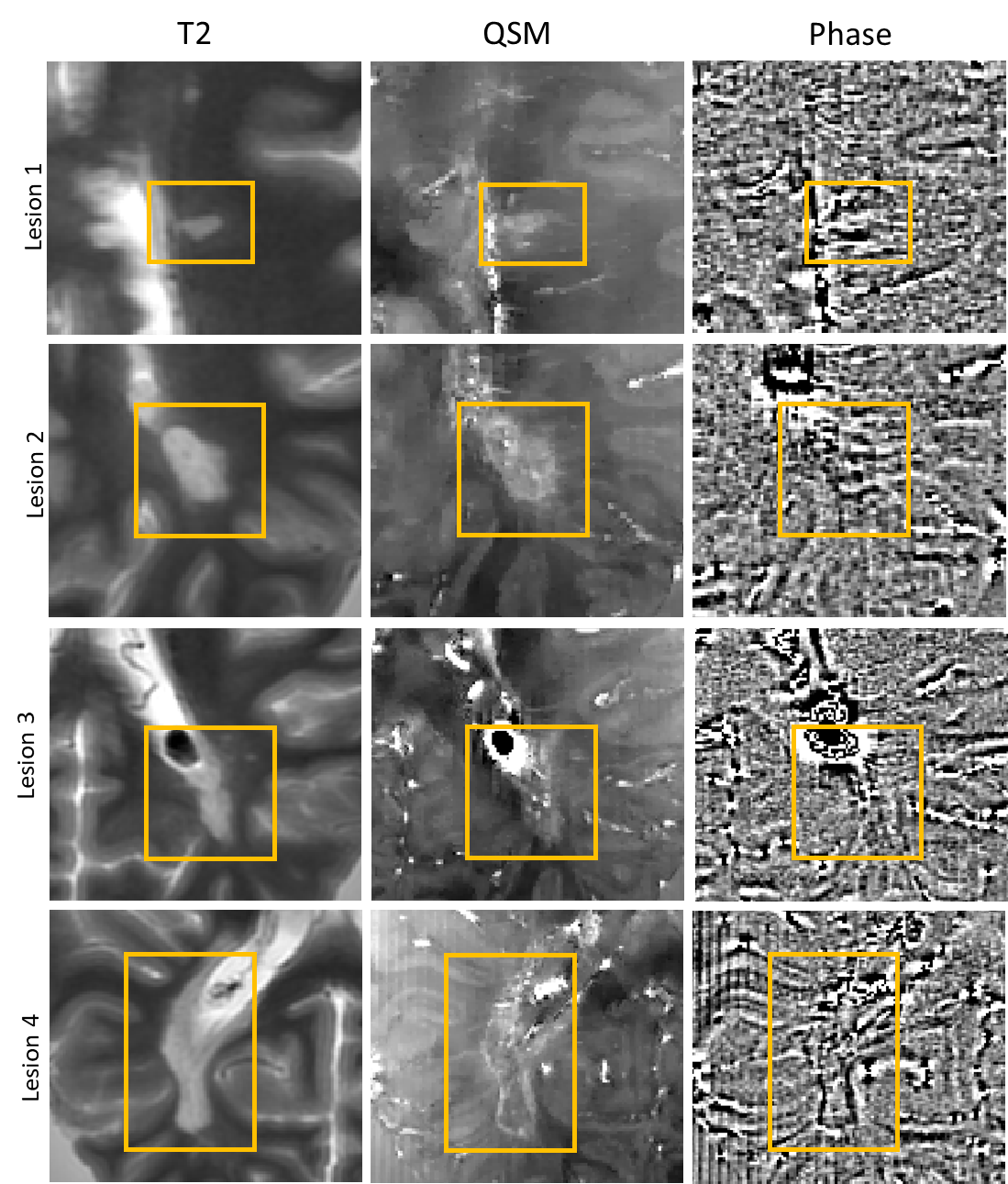
**

**
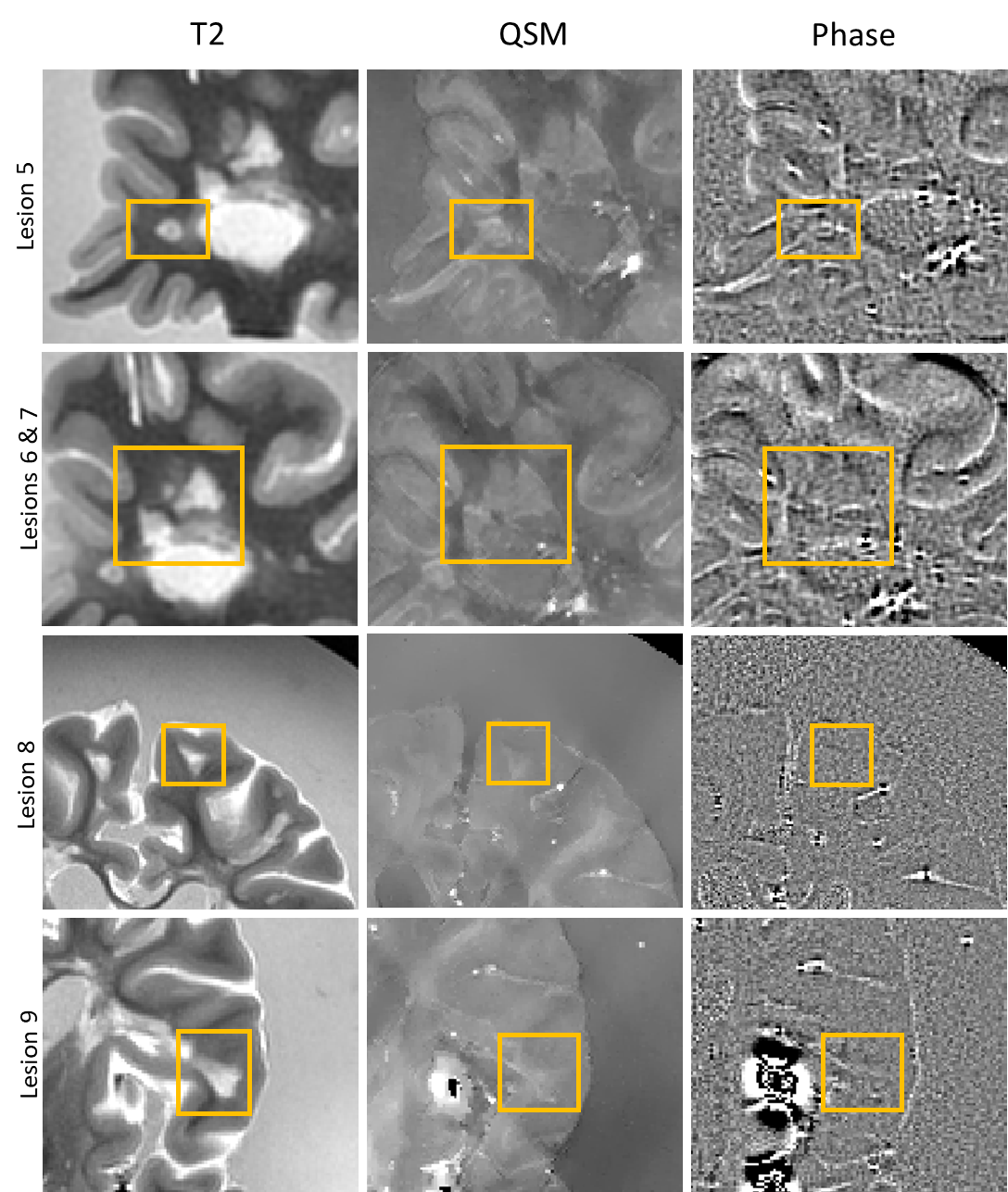
**

**
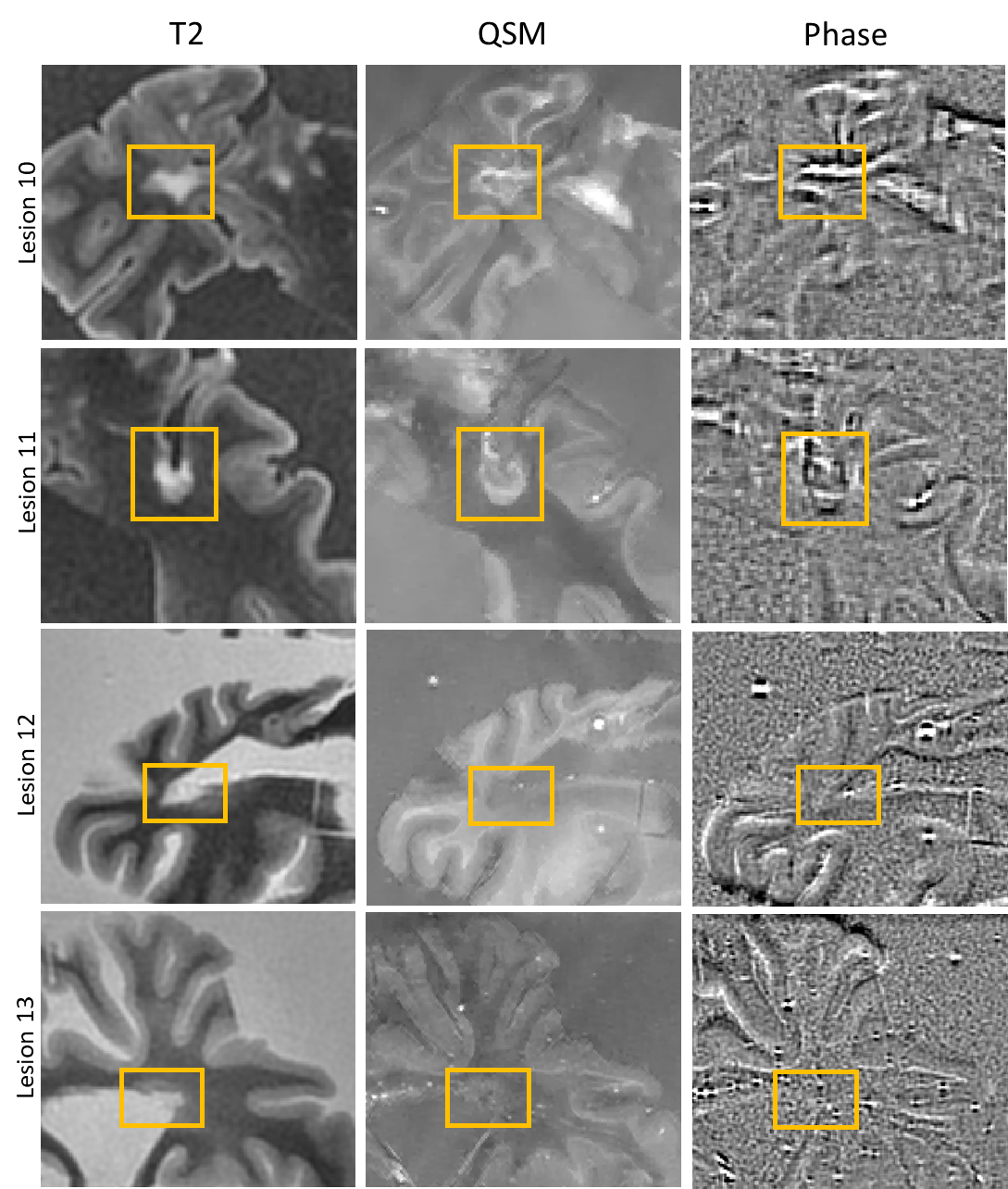
**

**
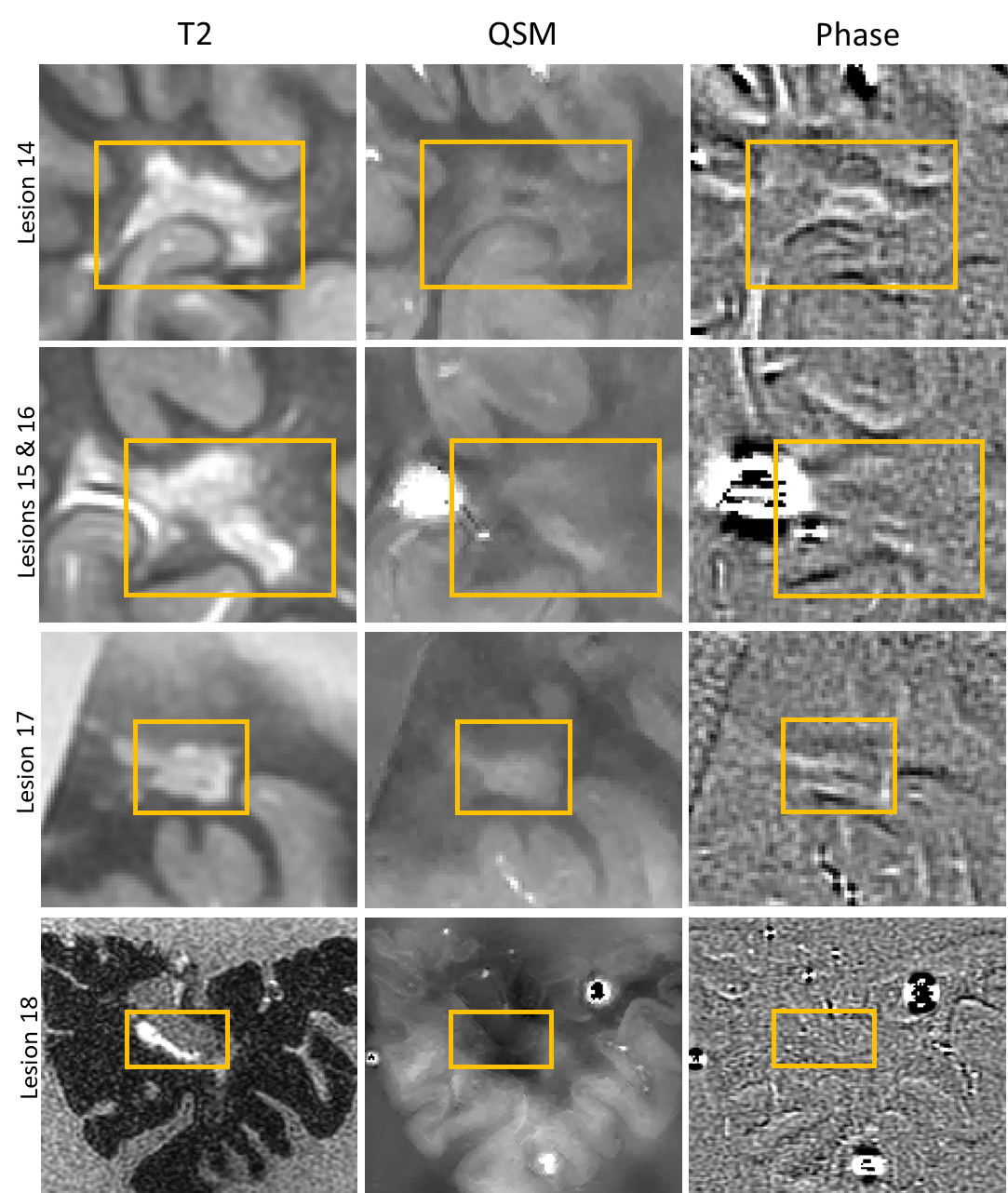
**

**
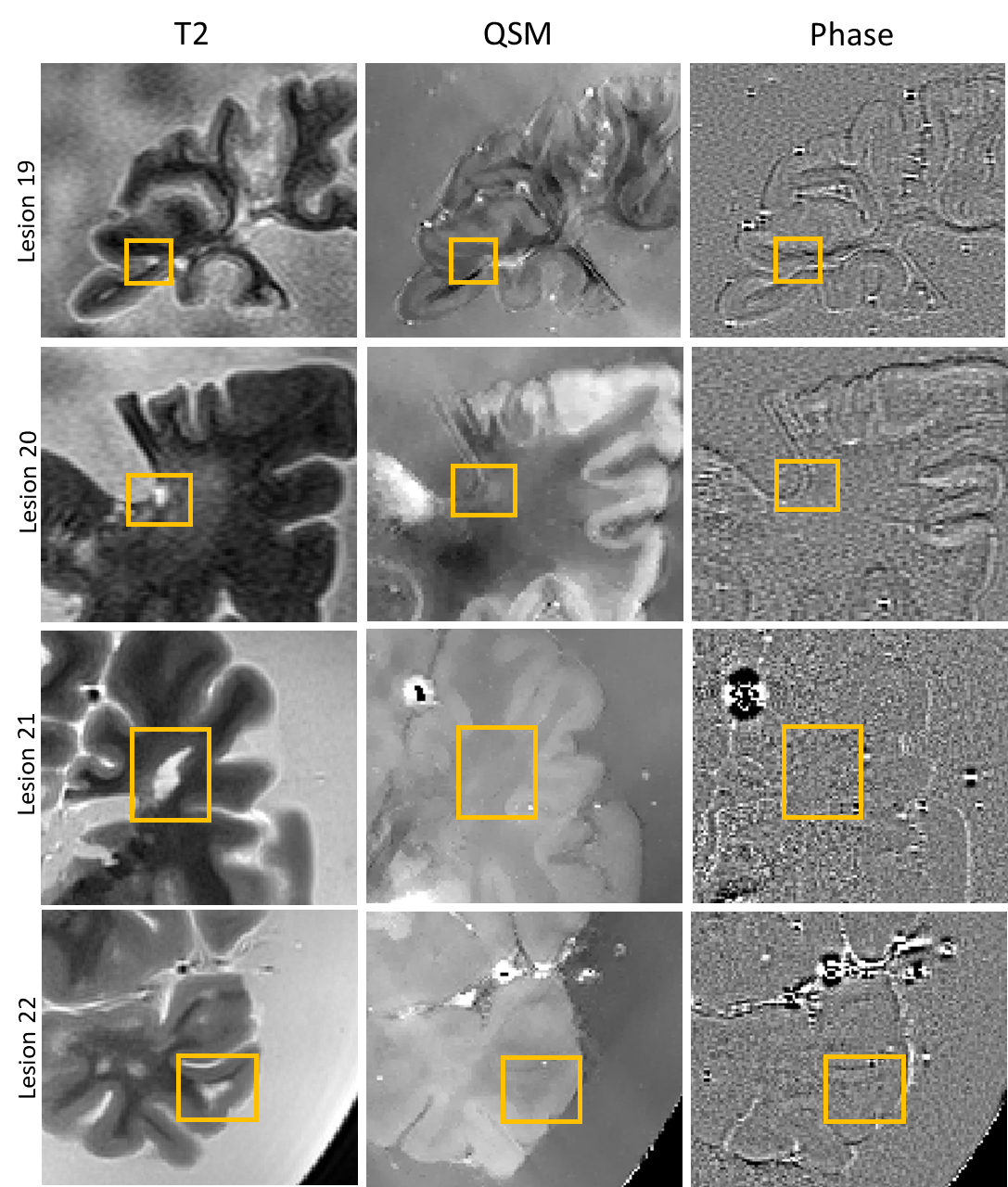
**

**
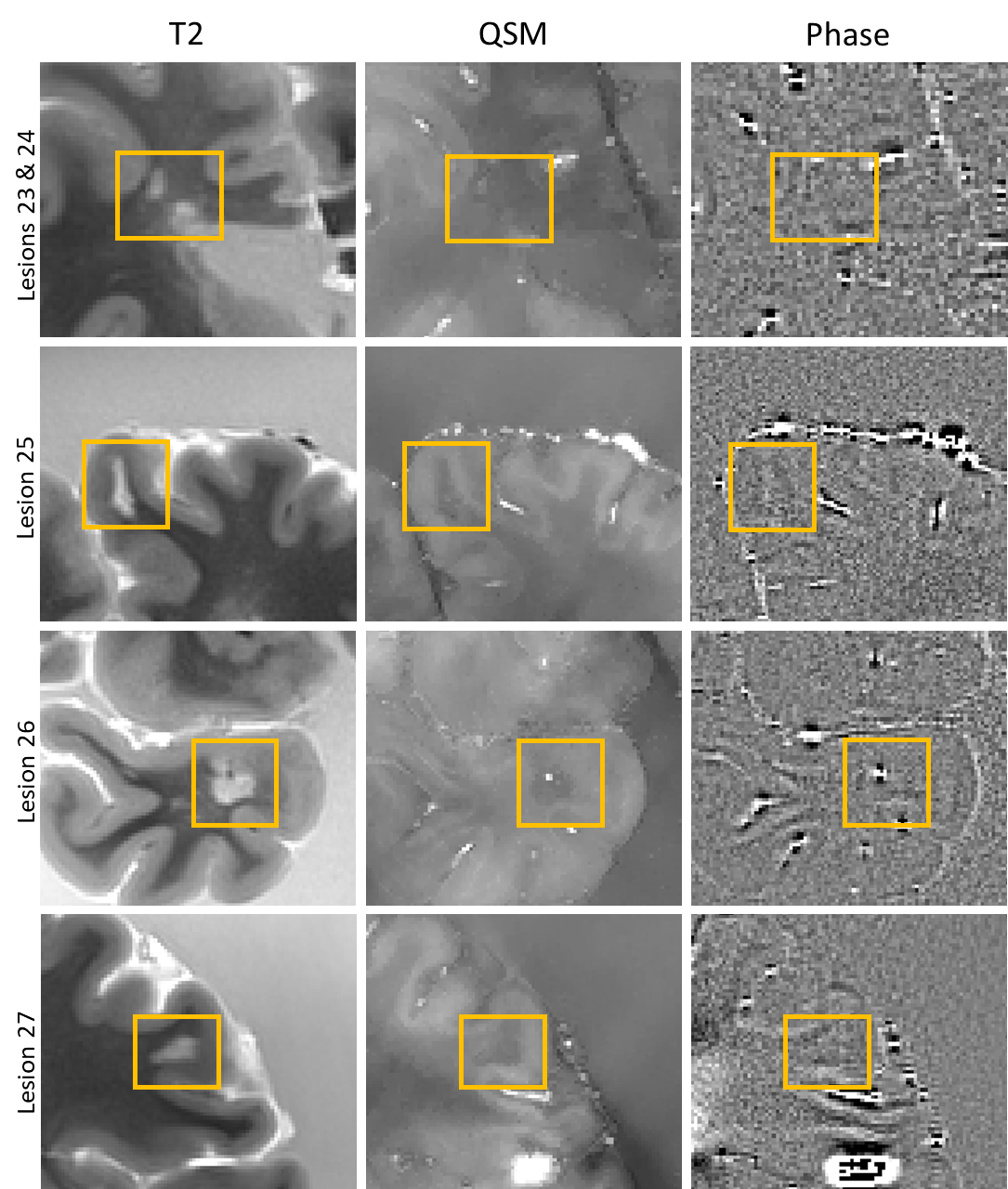
**

**
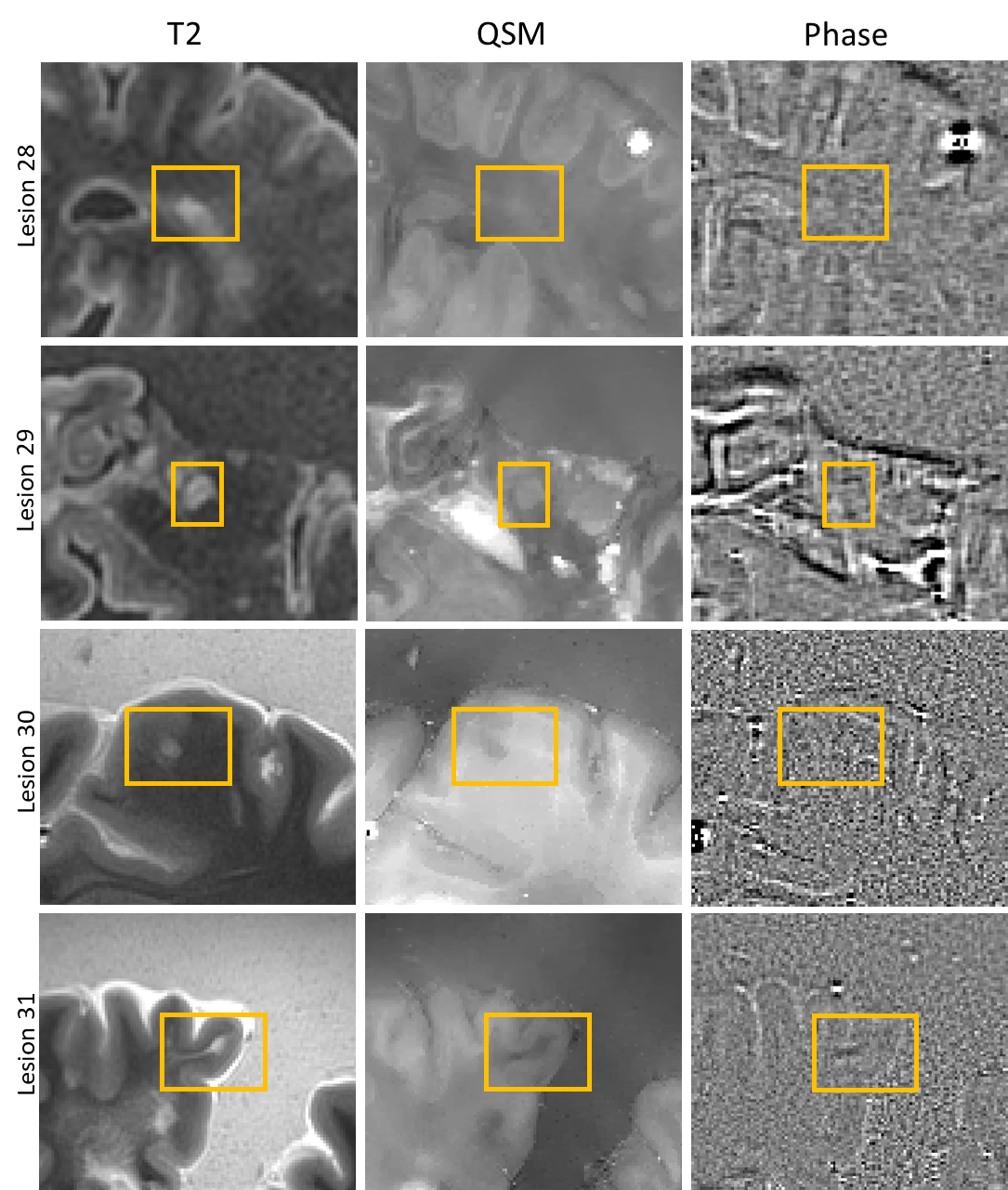
**

**
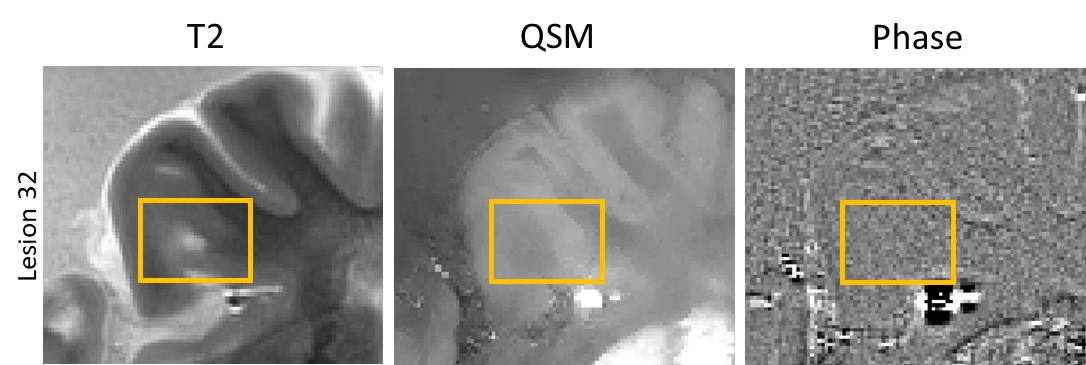
**

**Supplementary Figure 1. T2, Phase, and QSM of all 32 MS lesions.** Lesions 1 – 11 were classified as iron+ as determined by Perls’ staining while lesions 12 – 32 were classified as iron- as determined by Perls’ staining.
